# Not Feeling It: Modifiable Correlates of Anhedonia

**DOI:** 10.3390/bs16040533

**Published:** 2026-04-02

**Authors:** Marina F. Thomas, Gloria Mittmann, Marie Celine Dorczok, Verena Steiner-Hofbauer

**Affiliations:** Research Centre Transitional Psychiatry, University Hospital Tulln–NOE LGA, Faculty of Medicine, Karl Landsteiner University, 3430 Tulln, Austria; gloria.mittmann@kl.ac.at (G.M.);

**Keywords:** substance use, coping, problematic social media use, social media addiction, Internet addiction, mindfulness, depression

## Abstract

Background: Anhedonia denotes a reduced capacity of experiencing pleasure, which is often reported by individuals with psychiatric disorders such as depression and polysubstance use disorders. Since anhedonia is a critical factor influencing the well-being of psychiatric and general populations, it is important to investigate predictors of anhedonia. Method: We conducted a survey in *N* = 300 young adults aged 18 to 30 (*M* = 25.45, *SD* = 3.66). As predictors of anhedonia, we examined polysubstance use, problems with social media use, coping styles, and mindfulness. We controlled for age, gender, education, and the frequency of leisure activities. Results: Together, the predictors explained 20% of the variance in anhedonia. We found a positive association of polysubstance use with anhedonia, indicating that the more substances individuals consumed, the more anhedonia they reported. Problems with social media were not significantly related to anhedonia. Concerning coping styles, individuals with a more problem-focused coping style (e.g., planning) reported significantly lower levels of anhedonia, whereas emotion-focused coping (e.g., seeking social support) was unrelated to anhedonia. Mindfulness negatively correlated with anhedonia. Conclusions: The present study adds to research on behavioral and dispositional predictors of anhedonia and underlines the positive impact of mindfulness and problem-focused coping on anhedonia.

## 1. Introduction

Anhedonia, characterized by a diminished capacity to experience pleasure and pleasant emotions, represents a significant clinical feature across various psychiatric disorders, mainly dysthymia and depression but also schizophrenia or polysubstance use disorders (Kazdin, 2000; Trøstheim et al., 2020). Conceptually, anhedonia can be divided into two primary components: anticipatory anhedonia, referring to the inability to anticipate future pleasure, and consummatory anhedonia, which relates to reduced enjoyment during pleasurable experiences (Gard et al., 2007). This distinction underscores the complexity of anhedonia as a multidimensional phenomenon with implications for understanding both normal and pathological emotion processing. For example, Strauss et al. (2013) argue that in the context of schizophrenia, deficits are more pronounced in anticipatory pleasure.

Anhedonia is increasingly conceptualized as a dysfunction in reward processing rather than merely a reduction in experienced pleasure. Contemporary models distinguish multiple components of reward, including anticipation, motivation, effort, and consummatory enjoyment, which rely on partly dissociable neural and psychological mechanisms (Berridge & Robinson, 2003; Treadway & Zald, 2011). Disruptions in any of these components may contribute to anhedonia (Der-Avakian & Markou, 2012; Trøstheim et al., 2020). Neurobiological research links anhedonia to altered dopaminergic functioning and reduced responsivity in reward-related circuits, particularly within the ventral striatum and mesocorticolimbic pathways (Der-Avakian & Markou, 2012; Pizzagalli et al., 2009).

Importantly, reward processing is not solely a neurobiological phenomenon but is also shaped by patterns of behavioral engagement and environmental reward exposure. Everyday pleasurable experiences unfold across multiple domains, including social interaction, sensory experiences, hobbies, and food-related activities (Kringelbach & Berridge, 2010; Rizvi et al., 2015). Recent evidence suggests that these domains are embedded in ordinary, relational, and embodied contexts, highlighting the ecological and multidimensional nature of hedonic functioning (Mittmann et al., 2025). Furthermore, psychometric comparisons indicate that multidimensional and personalized measures such as the Dimensional Anhedonia Rating Scale (DARS) may capture nuanced variations in reward sensitivity beyond purely consummatory pleasure (Mittmann et al., 2026).

Taken together, these perspectives suggest that anhedonia reflects not only diminished pleasure experience but also altered engagement with rewarding activities in daily life. Consequently, modifiable psychosocial behaviors that influence exposure to, valuation of, or engagement with rewarding stimuli may be particularly relevant for understanding individual differences in anhedonic symptoms.

Anhedonia exhibits significant correlations with other psychological constructs. It is related to but distinct from neuroticism, depression, and guilt, and is associated with pleasureless introversion (Peterson & Knudson, 1983). These interconnections highlight the need for integrative approaches that take comorbid traits and overlapping symptom domains into account when designing diagnostic frameworks and treatment strategies. For instance, cognitive-behavioral therapies (CBT) targeting maladaptive thought patterns and behaviors have shown promise in alleviating anhedonia by enhancing engagement with rewarding activities (Craske et al., 2016). More recently, a randomized controlled trial found that a positive affect treatment led to greater improvements in clinical status and reward sensitivity than a CBT protocol focused on negative affect. Specifically, participants showed increased reward anticipation and responsiveness, as well as reduced anhedonia (Craske et al., 2023). Although several therapeutic approaches have demonstrated promise in alleviating anhedonia, important limitations remain. Many established treatments were not originally designed to directly target reward-processing deficits, and improvements in anhedonia are often secondary to broader symptom reduction (Craske et al., 2016; Rizvi et al., 2016). Even in interventions explicitly addressing positive affect or reward sensitivity, treatment effects on anhedonia are sometimes modest, and residual reward dysfunction may persist despite overall clinical improvement (Craske et al., 2023; Treadway & Zald, 2011). Moreover, most intervention studies focus on clinical samples, leaving open questions regarding the behavioral and dispositional factors associated with anhedonic symptoms in community populations (Trøstheim et al., 2020). A clearer understanding of naturally occurring, modifiable psychosocial correlates of anhedonia may therefore help refine transdiagnostic approaches targeting reward dysfunction and inform prevention strategies.

As a transdiagnostic feature, the inability to experience pleasure plays a critical role in the development, persistence, and treatment response of several mental health conditions. A recent review (Wong et al., 2024) found that anhedonia was significantly related to decreased health-related quality of life and functional outcomes in persons with major depressive disorder.

### 1.1. Polysubstance Use

Neurobiological findings suggest that reduced reward sensitivity, particularly diminished ventral striatum reactivity, plays a central role in anhedonia (Corral-Frías et al., 2015). A desensitization to rewarding stimuli may contribute to the blunted anticipation and experience of pleasure observed in affected individuals (Corral-Frías et al., 2015). Polysubstance use has been linked to alterations in reward processing, although effects appear to differ across substances. For example, chronic cannabis use has been associated with reduced neural responsiveness to natural rewards and alterations in dopaminergic signaling within reward-related circuits (Poyatos-Pedrosa et al., 2024). At the same time, some substances such as ketamine (Cao et al., 2019; Kwaśny et al., 2024), aticaprant, and psilocybin (Serretti, 2025) have been shown to alleviate anhedonia in controlled clinical trials. These contrasting findings highlight that substances may influence reward processing in different ways depending on pharmacological properties, patterns of use, and clinical context.

Beyond the effects of individual substances, research on polysubstance use suggests that the combined use of multiple substances is associated with greater psychological risk and broader patterns of polysubstance-related dysfunction (Elliott et al., 2019). However, the relationship between overall polysubstance exposure and anhedonic symptoms remains insufficiently understood. Examining the number of substances used may therefore provide an initial indicator of broader patterns of polysubstance-related reward dysregulation in community samples.

### 1.2. Problems with Social Media Use

Certain affordances of social media have been linked to changes in reward processing (Lakhan, 2025). This is because social media platforms are designed to deliver rapid and frequent rewards such as Likes or matches (Thomas et al., 2024). Excessive exposure to such fast digital rewards may overstimulate the reward system and contribute to a reduced sensitivity to slower-building and sustainable sources of pleasure. Over time, easy access to such rewards may undermine reward motivation and responsiveness to offline rewards (Gallagher et al., 2025). Thereby, uncontrolled use of social media may be associated with increased anhedonia. So far, neuroimaging research has shown that receiving social feedback such as Likes activates reward-related neural circuitry, including the ventral striatum (Sherman et al., 2016). This suggests that online interactions can provide reinforcing social rewards. However, whether problems with social media use are linked to anhedonia still warrants empirical investigation.

### 1.3. Coping

Coping strategies may play an important role in buffering against anhedonic symptoms. Contemporary coping research distinguishes between problem-focused coping (e.g., active problem-solving or planning), emotion-focused coping (e.g., seeking emotional support), and avoidant coping (e.g., disengagement or denial) (Carver, 1997). These strategies may differentially influence individuals’ engagement with potentially rewarding experiences. Problem-focused and other approach-oriented coping strategies may promote goal-directed behavior and sustained involvement in meaningful activities, thereby supporting hedonic functioning. In contrast, avoidant coping may reduce behavioral activation and limit opportunities for reward experience. Adaptive coping strategies, particularly active problem-solving and supportive social engagement, have been associated with reduced psychological distress and improved emotional functioning (Osei-Bonsu et al., 2014; Schetsche, 2022). By maintaining approach-oriented engagement rather than withdrawal, such strategies may help preserve exposure to rewarding stimuli and mitigate reward-related deficits. Consistent with this perspective, reduced engagement in adaptive, leisure-based coping strategies has been linked to higher levels of anhedonia and diminished benefit derived from pleasurable activities (Nagata et al., 2021). These findings suggest that the type and quality of coping strategy employed, rather than coping frequency alone, may be critical in understanding associations with anhedonia.

### 1.4. Mindfulness

Mindfulness involves purposefully maintaining attention on present experiences to enhance awareness of mental events, along with adopting an open, curious, and accepting attitude toward those experiences (Bishop et al., 2004). Several randomized controlled trials suggest that mindfulness-based interventions may reduce different anhedonic symptoms: An RCT with adults experiencing chronic stress found that an 8-week Mindfulness-Based Stress Reduction program significantly improved social anhedonia (Carlton et al., 2022). Similarly, mindfulness-based cognitive therapy was found to be effective in reducing anhedonia (Snaith et al., 1995), defined as consummatory pleasure (Cernasov et al., 2021; Rizvi et al., 2016). In another trial, an 8-week Mindfulness-Oriented Recovery Enhancement program enhanced reward responsiveness in veterans with chronic pain receiving long-term opioid therapy (Garland et al., 2023). Collectively, this evidence points to the potential of mindfulness to modulate specific aspects of anhedonia.

Given the complexity of anhedonia and its potential to mediate the relationship between mental health and quality of life, further research is essential to elucidate associations of anhedonia with malleable psychosocial aspects of life. This study focused on modifiable correlates rather than fixed traits to identify correlates that can potentially be addressed in interventions to alleviate anhedonia. Specifically, we examined polysubstance and media consumption, coping strategies, and mindfulness.

## 2. Methods

### 2.1. Participants

In winter 2024, a sample of 300 participants (57.3% female and one person of other gender) was recruited via a panel distribution by Gallup-Institut GmbH (Vienna, Austria). Participants were young adults aged 18 to 30 (*M* = 25.45, *SD* = 3.66). Concerning education, 40% reported high school as their highest educational level. Almost half (45.7%) were single. Most (79.7%) did not have children. The majority (67.3%) had never sought outpatient psychological treatment. Most (85.7%) had never sought inpatient treatment due to a psychological disorder. This study is part of a broader project on anhedonia (Mittmann et al., 2025; Mittmann et al., 2026).

### 2.2. Measures

We gauged the dependent variable anhedonia with the Dimensional Anhedonia Rating Scale (DARS) by Rizvi et al. (2015) because this measure is validated, and recommended for community samples (Mittmann et al., 2025). The DARS consists of 17 items assessing anhedonia across four domains: social activities, hobbies/pastimes (not social), favorite sensory experiences, and food/drinks. Participants are asked to write down two examples of each domain they find pleasurable and are then asked how much they would now enjoy these on a scale from 0 = “not at all” to 5 “very much”. As in prior studies (Mittmann et al., 2025; Rizvi et al., 2015), reliability was high (Cronbach’s α = 0.93). We re-coded all items so that high values indicate anhedonia and created a sum score (*M* = 29.64, *SD* = 11.24).

For measuring polysubstance use, we used items from Nightlife (Infodrog—Schweizerische Koordinations- und Fachstelle Sucht, 2023). In line with extant research (Elliott et al., 2019), participants were asked on how many days within the last 30 days individuals had consumed certain substances on a scale from 1 = “not at all” to 5 = “on more than 20 days”. Examples are THC, CBD, ecstasy, amphetamines, cocaine, and anabolic steroids (for the full list of 17 substances, see Appendix A). We did not aggregate the items because consumption patterns of substances may not be comparable: Some substances are consumed daily (e.g., ADHD medication), while others (e.g., LSD) are generally consumed more episodically. Simple aggregation would have underestimated the impact of substances that are consumed less frequently. Thus, we dichotomized each drug (use versus non-use) (Conway et al., 2013). Many participants (78%) in our sample consumed no substances, 12.7% only a single polysubstance, and the distribution showed a tail to the right with few individuals consuming many substances up until a maximum of 15. The prevalence of individual substances is reported in Table A1.

We created a sum score of the number of substances individuals had consumed in the last month by adding up all dichotomized substances but alcohol and tobacco. Alcohol and tobacco were assessed but not included in this index because their substantially higher prevalence and normative patterns of use differ from other substances and would have dominated the measure.^1^ As the number of substances consumed validly indicates substance use breadth (Connor et al., 2014; Smith et al., 2011) and our 13 items showed very high reliability (α = 0.96), we used the count variable for analysis.

Problems with social media use were assessed using the four-item Social Media Disorder Test (SMDT) (Wartberg et al., 2023), a brief screening instrument adapted from the Gaming Disorder Test. The SMDT has demonstrated a unidimensional factor structure, high internal consistency, and initial evidence of criterion validity in a sample of young adults. The items measured how often in the last 12 months individuals had problems controlling their social media activities, prioritized social media, kept using social media despite negative consequences, and had experienced problems due to intense social media use (*M* = 2.01, *SD* = 0.90, α = 0.82).

Coping styles were measured with the Brief COPE (Carver, 1997; Knoll et al., 2005). On a scale from 1 = “not at all” to 4 = “very much”, participants rated to what extent 28 coping strategies applied to them. Theoretically, there should be three dimensions (problem-focused, emotion-focused, and dysfunctional coping (Cooper et al., 2008). However, Since the factor structure of this validated measure can vary across samples (Krägeloh, 2011), it should be tested in each study (Carver, 1997; Kannis-Dymand et al., 2020). In line with prior research (Zelikovsky et al., 2007), we conducted a principal components analysis using oblimin rotation. It revealed eight factors in our sample of which only two were reliable (α > 0.65) and had more than two items. Therefore, we created the two variables problem-focused (e.g., making a plan; five items, *M* = 2.69, *SD* = 0.63) and emotion-focused (e.g., seeking social support; six items, *M* = 2.48, *SD* = 0.71) coping. Reliability was high in both problem-focused (α = 0.77) and emotion-focused (α = 0.85) coping.

We measured mindfulness with the well-validated Mindful Attention and Awareness Scale as translated by Michalak et al. (2011). Participants rated 15 statements on a six-point scale. Statements indicate mindless behaviors (e.g., doing things without paying attention). Items needed no recoding because the scale ranged from 1 = “almost always” to 6 = “almost never”, so high values indicate mindfulness (*M* = 3.72, *SD* = 0.88, α = 0.90).

As control variables, we asked for age in years, gender (female; male; other), education, and frequency of activities. We controlled for general differences in participants’ overall engagement in leisure activities to ensure that the observed associations of anhedonia with focal variables reflect differences beyond general activity levels. Leisure activity was measured with four items (meeting friends or family; social games; trips, activities; going out at night) on a four-point scale from “never” to “frequently”. We re-coded them so that higher values indicated higher activity (*M* = 2.76, *SD* = 0.57, α = 0.66).

### 2.3. Research Ethics

We report all data exclusions (if any) and we follow JARS (Appelbaum et al., 2018). The data is openly available on OSF (https://osf.io/emkc9/overview?view_only=615bfe62ad31487e891aad0b18b9993b; created 21 October 2025). The study was conducted in accordance with the Declaration of Helsinki. Approval from an ethics committee was not required because this study used only anonymous, non-interventional survey data, did not target vulnerable groups such as patients, and involved no deception (German Research Foundation, 2023). All participants were over 18 years old and gave informed consent.

### 2.4. Data Analysis

We analyzed the relative associations of all predictors with anhedonia within a single model because this takes into account the shared variance between predictors. We ran an ordinary least squares (OLS) multiple regression analysis controlling for age, gender, education, and frequency of activities.

## 3. Results

**Figure 1:**
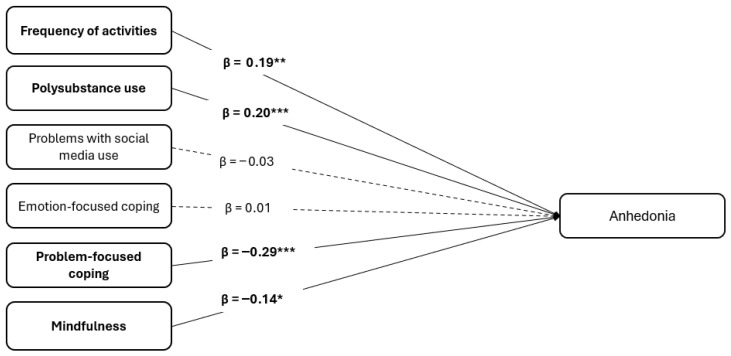
Modifiable Correlates of Anhedonia in Young Adults (*N* = 300). * *p* < 0.05, ** *p* < 0.01, *** *p* < 0.001.

Descriptive results can be found in Table 1. Results can be found in Table 2 and are visualized in Figure 1. The model (*F* [9, 288] = 9.22, *p* < 0.001) explained adj. R^2^ = 19.9% of anhedonia, indicating moderate but meaningful explanatory power. We report standardized coefficients because predictors had different scales. The number of substances was significantly related to anhedonia (*β* = 0.20, *p* < 0.001), suggesting that the more substances individuals consumed, the more anhedonia they reported. Problems with social media were unrelated to anhedonia, *β* = −0.03, *p* = 0.562. While emotion-focused coping was unrelated to anhedonia, *β* = 0.01, *p* = 0.861, problem-focused coping was negatively related to anhedonia, *β* = −0.29, *p* < 0.001. We found lower levels of anhedonia in individuals who reported a more problem-focused coping style. Mindfulness negatively correlated with anhedonia, *β* = −0.14, *p* = 0.011.

Concerning covariates, age (*β* = 0.02, *p* = 0.722), gender (*β* = −0.05, *p* = 0.316), and education (*β* = −0.11, *p* = 0.064) were unrelated to anhedonia. Only activity frequency showed a negative association with anhedonia, *β* = −0.19, *p* = 0.001.

## 4. Discussion

The present study explored behavioral and dispositional predictors of anhedonia in a community sample of young adults. We examined the roles of polysubstance use, problems with social media use, coping styles, and mindfulness while controlling for age, gender, education, and leisure activity frequency. Together, the predictors explained 20% of the variance in anhedonia.

Our findings revealed that higher polysubstance use was significantly associated with greater levels of anhedonia. In contrast, problems with social media use showed no significant relationship with anhedonia. Regarding coping styles, a more problem-focused coping approach was linked to lower anhedonia, whereas emotion-focused coping was unrelated to anhedonia. Finally, more mindfulness was associated with lower levels of anhedonia.

Our results align with existing literature suggesting that polysubstance use may disrupt reward-related brain functioning and contribute to blunted affective responses (Der-Avakian & Markou, 2012; Poyatos-Pedrosa et al., 2024). The observed association may reflect a complex interplay between polysubstance use and anhedonia. However, given the cross-sectional design, no conclusions regarding directionality can be drawn. Prior research suggests that reduced reward sensitivity may increase vulnerability to polysubstance use, while polysubstance use may also be linked to elevated anhedonic symptoms (Corral-Frías et al., 2015; Pizzagalli et al., 2009). While certain psychoactive substances like ketamine or psilocybin have shown short-term effects in reducing anhedonia under controlled conditions (Kwaśny et al., 2024; Serretti, 2025), our findings show that consuming a higher number of substances is negatively associated with hedonic capacity in everyday contexts. Our polysubstance use variable did not include alcohol and tobacco, but the findings remain robust when alcohol and tobacco are included.

The absence of a significant association between problems with social media use and anhedonia does not provide support for theoretical accounts suggesting that frequent exposure to fast digital rewards (e.g., likes, notifications) may desensitize the reward system and reduce motivation for naturally rewarding offline stimuli (Cangelosi et al., 2025; Lakhan, 2025; Yu et al., 2023). Although problems with social media use may be related to other domains of mental health, its association with reward-related deficits appears to be limited.

In addition, the frequency of leisure activities showed a significant negative association with anhedonia. Individuals who reported engaging more frequently in social and recreational activities also reported lower levels of anhedonic symptoms. This finding aligns with theoretical models that conceptualize anhedonia partly as a reduction in behavioral engagement with rewarding environments. Regular participation in social and leisure activities may increase exposure to potentially rewarding experiences and thereby help maintain hedonic functioning. This interpretation is consistent with behavioral activation frameworks, which emphasize increasing engagement with meaningful and pleasurable activities as a mechanism for reducing anhedonic symptoms and improving mood (Craske et al., 2016).

The observed relationship between problem-focused coping and reduced anhedonia is consistent with theoretical accounts emphasizing the protective role of active and goal-directed coping strategies in mental well-being (Craske et al., 2016). Problem-focused coping typically involves behaviors such as planning and active problem solving that aim to directly address stressors and maintain engagement with ongoing activities (Biggs et al., 2017; Carver, 1997). Maintaining such engagement may increase exposure to potentially rewarding experiences and thereby support hedonic functioning. In contrast, emotion-focused coping was not significantly related to anhedonia in the present study. Importantly, emotion-focused coping encompasses a broad range of strategies beyond seeking social support, including emotional expression, acceptance, or cognitive reframing (Carver, 1997). While these strategies may help regulate distress, they do not necessarily increase behavioral engagement with potentially rewarding environments.

Lastly, higher levels of mindfulness were significantly associated with reduced anhedonia. Prior research has shown that mindfulness-based interventions can improve aspects of hedonic functioning, including social anhedonia (Carlton et al., 2022) and reward responsiveness in chronic pain patients (Garland et al., 2023). These effects have been attributed to mechanisms such as enhanced attentional control, increased present-moment awareness, and reduced experiential avoidance, which may increase sensitivity to naturally occurring rewarding experiences (Bogaert et al., 2023). While the present findings cannot speak to causal mechanisms or intervention efficacy, the observed association aligns with theoretical accounts proposing that enhanced awareness and behavioral engagement may support hedonic functioning. Relatedly, intervention research has shown that approaches such as positive affect treatment (Craske et al., 2023) and behavioral activation in adolescents (Webb et al., 2024) can reduce anhedonia and increase engagement with rewarding activities. These findings highlight the relevance of behavioral engagement for restoring reward sensitivity, while also pointing to the importance of tailoring interventions to individual motivational dynamics.

Taken together, our findings reinforce the conceptualization of anhedonia as a multidimensional construct shaped by behavioral and cognitive–affective processes. The differential associations observed across polysubstance use, coping, and mindfulness underline the importance of considering both risk and resilience factors when assessing anhedonic symptoms in community samples.

### 4.1. Limitations

Several limitations should be considered when interpreting the present findings. First, the cross-sectional design precludes conclusions regarding directionality or causality. Although associations between psychosocial variables and anhedonia were observed, longitudinal and experimental studies are required to clarify temporal relationships and underlying mechanisms. Second, all constructs were assessed via self-report measures, which may be subject to recall bias and shared method variance. Future research incorporating behavioral or neurobiological indices of reward processing could provide a more comprehensive understanding of the mechanisms underlying anhedonia. Third, the study was conducted in a community sample of young adults with generally low levels of polysubstance use and problems with social media use. This may limit the generalizability of findings to clinical populations or samples with higher levels of behavioral dysregulation. Finally, while the study examined several psychosocial correlates of anhedonia, reward sensitivity and neural reward processing were not directly assessed. Thus, interpretations linking the observed associations to reward-related mechanisms remain theoretical and should be tested in future research.

### 4.2. Future Directions

Future research should employ experimental and longitudinal methodologies to better understand causal and temporal pathways between polysubstance use, coping mechanisms, mindfulness, and anhedonia. Experimental interventions targeting mindfulness and problem-focused coping strategies could help determine their potential as therapeutic approaches. Investigating individual differences, such as susceptibility to reward deficits or specific patterns of social media (over-)use, could further refine our understanding of anhedonia’s behavioral correlates. Lastly, neuroimaging studies could complement self-reported data to elucidate the underlying neural mechanisms linking these variables.

## 5. Conclusions

This study contributes to the growing body of literature on anhedonia by identifying modifiable behavioral and psychological factors associated with its severity. Our findings highlight polysubstance use as a risk factor, whereas problem-focused coping and mindfulness emerge as protective factors. The lack of an association between problems with social media use and anhedonia challenges common narratives about digital engagement and emotional blunting. Future research should build upon these insights to inform the development of targeted interventions aimed at mitigating anhedonia’s impact on mental well-being.

## Figures and Tables

**Table 1:** Descriptive results for continuous variables.

Variable	Mean	SD
Age	25.45	3.66
Frequency of Activities	2.76	0.57
Polysubstance Use	0.80 (*Mdn* = 0)	2.69
Problems with Social Media Use	2.01	0.90
Emotion-focused Coping	2.48	0.63
Problem-focused Coping	2.69	0.71
Mindfulness	3.72	0.88
Anhedonia	29.64	11.24

**Table 2:** Regression Table.

Predictor	b	SE	beta	t	p
Age	0.07	0.18	0.02	0.36	0.722
Gender	−1.21	1.21	−0.05	−1.00	0.316
Education	−1.03	0.55	−0.11	−1.86	0.064
Frequency of Activities	−3.72	1.09	−0.19	−3.42	0.001 **
Polysubstance Use	−0.83	0.23	0.20	3.65	<0.001 ***
Problems with Social Media Use	−0.42	0.73	−0.03	−0.58	0.562
Emotion-focused Coping	0.17	0.97	0.01	0.18	0.861
Problem-focused Coping	−5.15	1.07	−0.29	−4.82	<0.001 ***
Mindfulness	−1.80	0.70	−0.14	−2.56	0.011 *

*Note.* * *p* < 0.05, ** *p* < 0.01, *** *p* < 0.001.

## Data Availability

The data presented in this study are openly available in [OSF] at [https://osf.io/emkc9/overview?view_only=615bfe62ad31487e891aad0b18b9993b; created 21 October 2025].

## Note

1 Additionally including alcohol and tobacco in the index did not change any of the effects (for additional analyses see: https://osf.io/emkc9/overview?view_only=615bfe62ad31487e891aad0b18b9993b; created 21 October 2025).

## Appendix A

List of 17 substances: “methamphetamine”, “LSD/Psylos”, “ketamine”, “anabolic steroids”, “amphetamines”, “ADHD medication”, “poppers”, “potency agents”, “benzodiazepines”, “ecstasy”, “opioids”, “laughing gas”, “cocaine”, “THC cannabis”, “CBD cannabis”, “alcohol” “tobacco/products containing nicotine”.

As shown in Table A1, the use of most individual substances was very rare. Conducting separate analyses for each substance would therefore have resulted in very small cell sizes and unstable estimates.

**Table A1:** List of substances and number and percentages of use.

Substance	n (Use)	%
Alcohol	197	65.7
Tobacco/Nicotine	89	29.7
THC cannabis	31	10.3
CBD cannabis	20	6.7
Ecstasy	18	6.0
Cocaine	12	4.0
Amphetamines	20	6.7
LSD/Psylos	12	4.0
Ketamine	12	4.0
Laughing gas	11	3.7
Poppers	13	4.3
Methamphetamine	13	4.3
Opioids	13	4.3
Potency agents	15	5.0
Benzodiazepines	18	6.0
ADHD medication	15	5.0
Anabolic steroids	17	5.7

*Note.* Values indicate the number and percentage of participants reporting use of each substance on at least one day in the past 30 days. Substances were not mutually exclusive.
